# Group Selection and Contribution of Minority Variants during Virus Adaptation Determines Virus Fitness and Phenotype

**DOI:** 10.1371/journal.ppat.1004838

**Published:** 2015-05-05

**Authors:** Antonio V. Bordería, Ofer Isakov, Gonzalo Moratorio, Rasmus Henningsson, Sonia Agüera-González, Lindsey Organtini, Nina F. Gnädig, Hervé Blanc, Andrés Alcover, Susan Hafenstein, Magnus Fontes, Noam Shomron, Marco Vignuzzi

**Affiliations:** 1 Institut Pasteur, Viral Populations and Pathogenesis Unit, CNRS UMR 3569, Paris, France; 2 Institut Pasteur, International Group for Data Analysis, Paris, France; 3 Sackler Faculty of Medicine, Tel Aviv University, Tel Aviv, Israel; 4 Centre for Mathematical Sciences, Lund University, Lund, Sweden; 5 Institut Pasteur, Lymphocyte Cell Biology Unit, CNRS URA 1960, Paris, France; 6 Division of Infectious Diseases, Pennsylvania State College of Medicine, Hershey, Pennsylvania, United States of America; University of Pittsburgh, UNITED STATES

## Abstract

Understanding how a pathogen colonizes and adapts to a new host environment is a primary aim in studying emerging infectious diseases. Adaptive mutations arise among the thousands of variants generated during RNA virus infection, and identifying these variants will shed light onto how changes in tropism and species jumps can occur. Here, we adapted Coxsackie virus B3 to a highly permissive and less permissive environment. Using deep sequencing and bioinformatics, we identified a multi-step adaptive process to adaptation involving residues in the receptor footprints that correlated with receptor availability and with increase in virus fitness in an environment-specific manner. We show that adaptation occurs by selection of a dominant mutation followed by group selection of minority variants that together, confer the fitness increase observed in the population, rather than selection of a single dominant genotype.

## Author Summary

When RNA viruses replicate, they do so with a high rate of error; hence, their populations are not composed of a single genotype, but of a swarm of different, yet related, genomes. This mutant spectrum has been described as the viral quasispecies, and its composition has important consequences for evolution, adaptation and emergence. In this study, we analysed adaptation in fine detail thanks to the use of the deep sequencing, and we determined the adaptative pathway of a model RNA virus, Coxsackievirus B3, to a new environment, A549 cells. Our results demonstrate that adaptation occurred in response to a differential expression of the virus receptors in the new cellular environment, compared to the former. Our experiments and mathematical analyses established that the corresponding increase in fitness resulted from the selection and contribution of a group of genotypes, including low frequency variants, and not to the effect of a single, dominant genome. Our work underscores the importance of considering group effects when studying RNA virus biology and evolution.

## Introduction

The extreme mutation rates of RNA viruses and the highly diverse populations they generate in few replication cycles are considered the basis for their rapid adaptation to new environments [1,2]. Such adaptive steps result in the emergence of new variants capable of escaping immune responses, resisting antiviral approaches, altering tissue tropism or crossing species barriers. In the past, experimental evolution of viruses in different host environments has proven to be a useful tool in quantifying fitness increases and the dynamics of adaptation. By classic sequencing techniques, some of the key genetic determinants responsible have been identified [3,4], but until the advent of deep sequencing, analysis of the mutational composition of RNA virus populations was hampered by lack of depth of sequence coverage. The potential to describe the whole virus mutant spectrum and detect variants that otherwise would be overlooked by conventional sequencing is fundamental to studying virus evolution and understanding emergence [5]. Recent work shows that deep sequencing can identify the emergence of escape mutants in experimental and clinical samples [6,7], and can be used to characterize the entire mutant spectrum of a virus population [8].

One of the goals in the field of emerging infectious diseases is to determine whether adaptation to novel hosts (species, tissues or cell types) can be identified for a recently introduced pathogen that is confronted with a less than optimal host environment [9–11]. Viruses are well-suited for studying adaptation and evolution for several reasons: i) high mutation rates ii) short generation time and iii) large population sizes. We used Coxsackie virus B3 (CVB3) as a model, since the genetics of this virus and the interactions between the cell receptors and viral capsid proteins (VP1, VP2 and VP3) are well characterized. CVB3 enters the cell through a primary receptor, the Coxsackie and Adenovirus Receptor (CAR) [12], while certain strains may use as co-receptor the Decay Accelerating Factor (DAF) [13,14], also known as CD55. To study expansion of host tropism, we passaged virus in two cellular environments, a highly permissive one and a less permissive one. By deep sequencing longitudinal samples of experimentally evolved populations, we identify the emergence of host environment-specific mutations undergoing positive selection. We show that Coxsackie virus adapts differently to two cell types according to receptor and co-receptor availability in a multi-step adaptation sequence that involves group selection of minority variants. Importantly, we reveal the significant contribution of several minority variants to the overall fitness of the entire population. Our results underscore the importance of characterizing RNA virus quasispecies during adaptation and virus evolution.

## Results

### Increase of fitness and genetic diversity of CVB3 during adaptation to permissive and less permissive host environments

**Fig 1:**
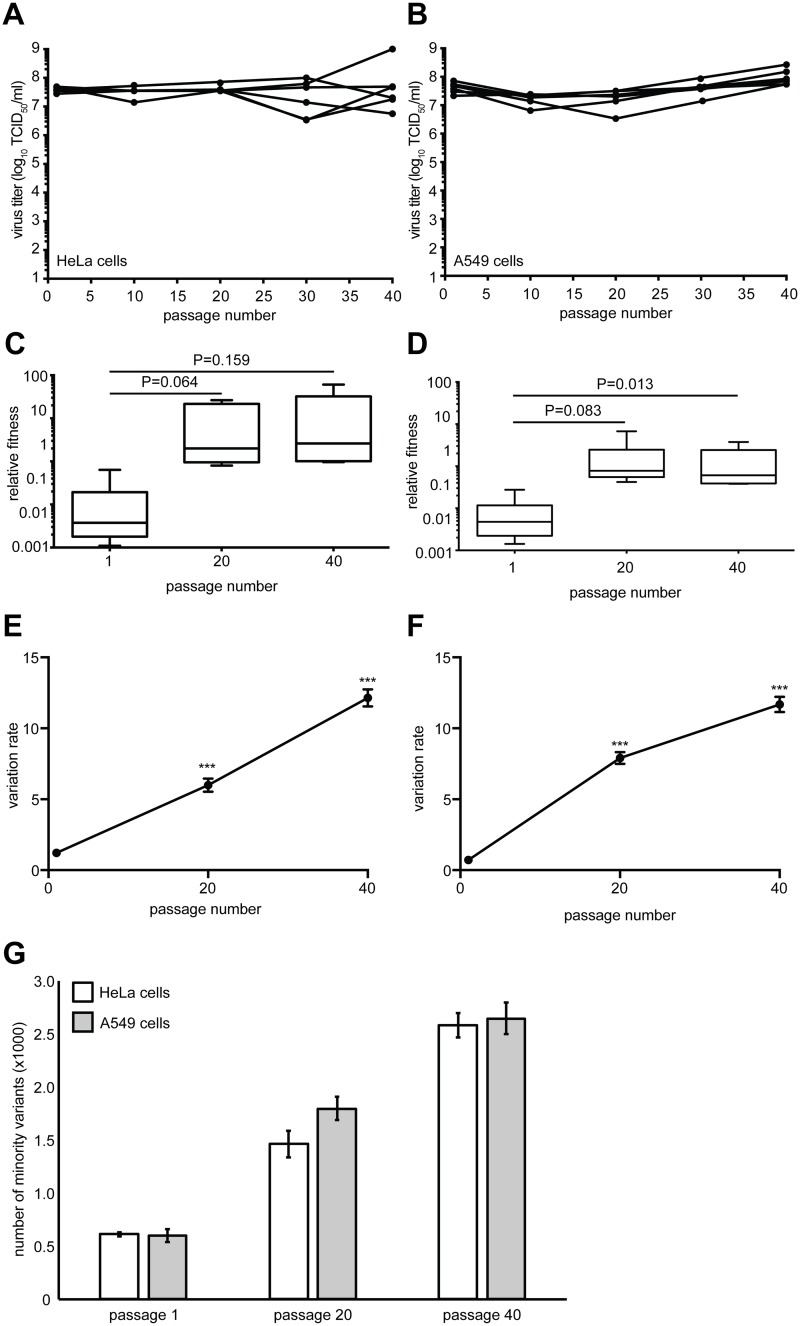
Virus fitness and genetic diversity increases during serial passage in both HeLa and A549 cells. HeLa (A) or A549 (B) cells were infected with CVB3 for 40 serial passages (x-axis) and virus progeny titers (y-axis) were determined for each of six replicate series. The relative fitness of each population from the HeLa (C) and A549 (D) series was compared to a non-passaged neutral competitor. Box plots show mean values and S.E.M., bars indicate minimum and maximum values, p values are indicated, student's paired t test, n = 6. The genetic diversity of each replicate was determined for passage 1, 20 and 40 populations in HeLa (E) and A549 (F) cells by calculating the variation rates at every nucleotide position. The mean variation rates ± S.E.M. are shown. ***p<0.0001, Mann Whitney test, n = 6. (G) The total number of minority variants in passage 1, 20 and 40 HeLa and A549 populations found within the P1 structural protein-coding region. Mean ± S.E.M. are shown. Differences between passage numbers are all significant, p<0.0001; no significant differences between cell type at each passage number, student's paired t test, n = 12.

To monitor the evolution of CVB3 towards novel and less permissive host environments, we selected human lung A549 cells, which gave similar final virus yields as the highly permissive HeLa cells, but after two days rather than one day of infection. CVB3 was thus serially passaged 40 times in six biological replicate series in both cell types. Virus yields were constant throughout the passage series suggesting that no significant genetic drift or accumulation of detrimental mutations through population bottlenecking had occurred (Fig 1A and 1B). The time required to reach peak titers was reduced in A549 cells over the first ten passages from 48 hours to 24 hours, suggesting that fitness increases and adaptation occurred in this novel environment. We measured the relative fitness of the passaged viruses by competing the passage 1, 20 and 40 populations from each replicate with a genetically marked neutral CVB3 virus in a quantitative fitness assay [15]. Increases in fitness were observed in both cell types, with the most significant increase found by passage 40 in A549 cells (Fig 1C and 1D), suggesting that adaptation had occurred in this less permissive cell type.

Whole-genome deep sequencing of these populations revealed significant increases in overall genetic variation, throughout the genome, between the 1st, 20th and 40th passages in both HeLa (Fig 1E) and A549 (Fig 1F) cells (p<0.0001). The total number of minority variants within the P1 structural coding region also significantly increased over the passage series (Fig 1G), yet there were no significant differences between the numbers observed in Hela and A549 cell passage. The vast majority (>98%) of these variants were low frequency (<1.0% of the total population), suggesting that moderate genetic drift, rather than positive selection, was responsible for most of this variance. The increase in diversity in both cell types is likely the result of general population expansion in sequence space, since all passage series were started from homogenous *in vitro* transcribed RNA derived from an infectious clone. The data also confirmed that severe bottlenecking did not occur during the passage series where expansion of diversity should have been stalled or lost.

### The mutations that arise from adaptive passage map to receptor binding sites in a cell-type specific manner

Although the HeLa and A549 cell passage series presented similar mean genetic diversity at passage 40 (Fig 1E–1G, P = 0.314), we mined the deep sequence data for all minority variants above 1% frequency that might explain adaptation in each condition (Table 1). In both cases, several mutations recurring in multiple replicates mapped to residues known to be part of the CAR receptor and DAF co-receptor footprints [16]; however, the distribution of these mutations was different for the HeLa- and A549-adapted viruses. In HeLa cells, the most abundant variants involved mutations strictly in the CAR footprint (VP1-259, 2–7% frequency), in the CAR/DAF shared footprint (VP2-138, 20–80%), and in the DAF footprint (VP3-234, 2–73% frequency; VP3-63, 3–14% frequency). In contrast, viruses passaged in A549 cells presented no variants strictly involved in the CAR footprint. Instead, in addition to CAR/DAF residue VP2-138 (2–81% total), a larger cluster of exclusively DAF-footprint variants were identified (VP3-63, 3–35% frequency; VP3-234, 3–82% frequency and VP1-271, 2–13% frequency). Strikingly, the most abundant mutation in every replicate passaged in A549 cells, and not observed in HeLa cell passage, was VP3-76 (61–95% total). This residue is not known to participate in either footprint.

**Table 1:** Minority variants (>1% threshold) present in the passage 40 populations of the six CVB3 replicates, passaged in either HeLa or A549 cells.

		replicate						receptor
	capsid protein	1	2	3	4	5	6	footprint
**HeLa**	VP1	K259M						CAR
				K259N	K259N			
		K259R		K259R	K259R	K259R	K259R	
	VP2	D138E	D138E	D138E	D138E	D138E	D138E	CAR/DAF
		D138G	D138G			D138G	D138G	
		D138N	D138N	D138N	D138N			
	VP3			N63H	N63H		N63H	DAF
		Q234K	Q234K					
						Q234R	Q234R	
**A549**	VP1	T271P	T271P					DAF
	VP2	D138E		D138E	D138E	D138E	D138E	CAR/DAF
		D138G	D138G	D138G	D138G		D138G	
		D138N					D138N	
		K166N						
		K166E						
	VP3	N63D	N63D	N63D	N63D			DAF
			N63H		N63H		N63H	
		N63H	N63S					
		N63Y	N63Y	N63Y				
		Q234K	Q234K	Q234K	Q234K	Q234K	Q234K	
			Q234L			Q234L		
			Q234R		Q234R		Q234R	
		E76G	E76G	E76G	E76G	E76G	E76G	unknown

The amino acid changes for each variant are listed according to capsid protein (VP1, VP2 or VP3), and their involvement in interacting with either or both receptors is indicated (receptor footprint).

### Permissive and less permissive cell environments present different expression profiles of the primary and secondary receptors

**Fig 2:**
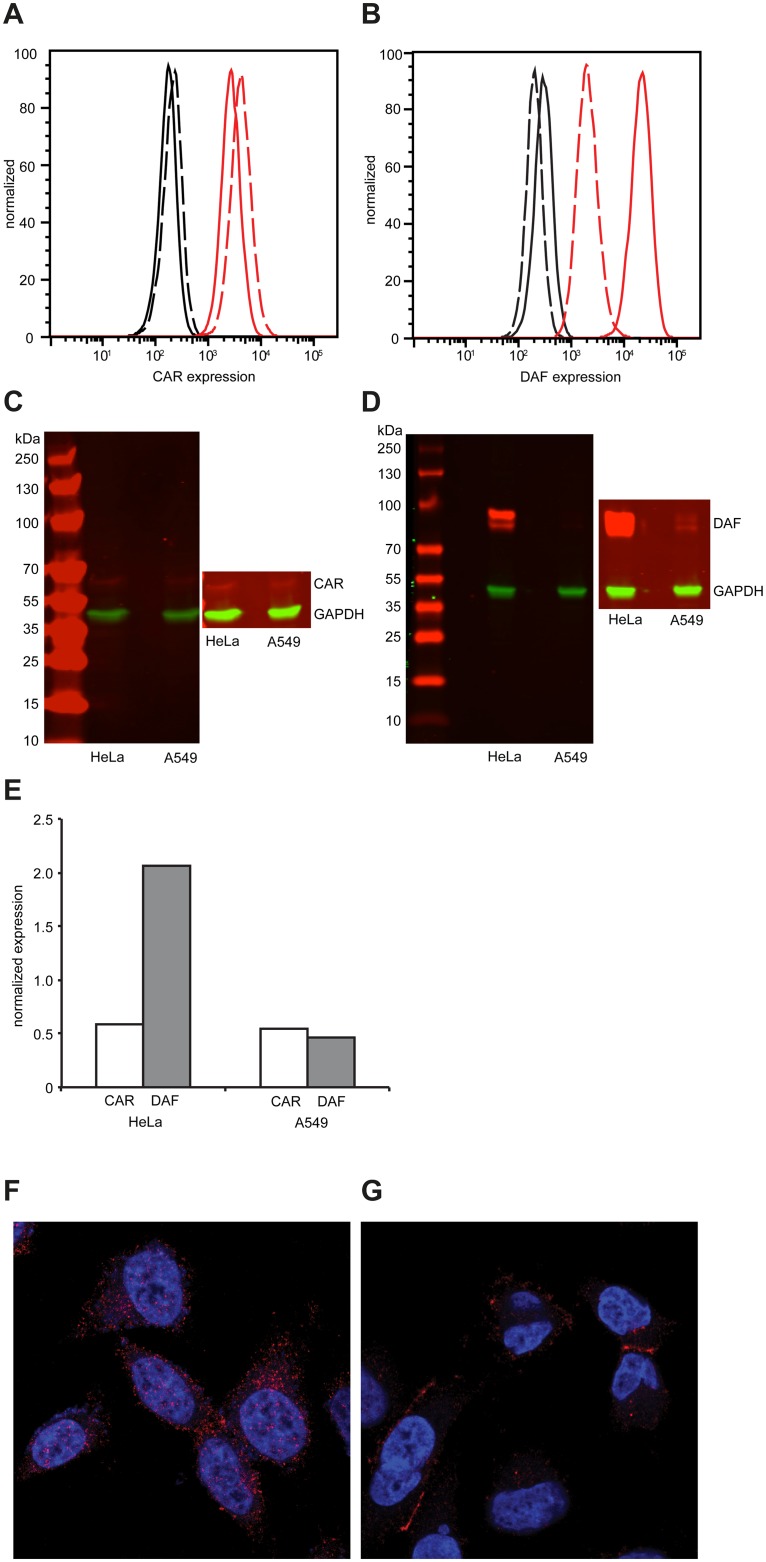
Differential expression of CAR and DAF in HeLa and A549 cells. (A-B) Expression of CAR (A) and DAF (B) by flow cytometry in HeLa cells (solid line) or A549 cells (dashed line). Fluorescence of cells in absence of antibody (black) or in presence of CAR- or DAF-specific antibody (red) is shown. Figures are representative of three independent experiments. (C-D) Expression of CAR (C) and DAF (D) by Western blot analysis of HeLa or A549 cell extracts, revealed by CAR-, DAF- and GAPDH-specific antibodies. Molecular weight markers are indicated on the left, super-exposed section of each figure is included to reveal the presence of CAR and the two isoforms of DAF in each sample. Figures are representative of two independent experiments. (E) The relative expression of CAR and DAF with respect to GAPDH was quantified by imaging quantification using ImageJ. Localization of CAR, in red, in HeLa (F) or A549 (G) by confocal microsocopy. Nuclear (blue) staining was done with DAPI.

Considering these CAR/DAF-specific differences, we hypothesized that expression levels of these molecules must vary between these cells. There were no significant differences in CAR expression in either cell by both flow cytometry (Fig 2A) or Western blot (Fig 2C). On the other hand, while DAF was very highly expressed in HeLa cells (Fig 2B and 2D), expression was one order of magnitude lower in A549 cells by flow cytometry (Fig 2B) and barely detectable by Western blot (Fig 2D). Normalized quantification of Western blot signals revealed that DAF expression was 4-fold higher in HeLa than in A549 cells (Fig 2E). To characterize the localization of CAR and DAF in these cell types, confocal microscopy was performed. In HeLa cells, CAR (Fig 2F) and DAF (S1 Fig) were expressed throughout the surface of the cell. Interestingly, in A549 cells CAR predominantly localized at cell-to-cell contacts (Fig 2G), while DAF was diffused throughout the surface of the cell (S1B Fig), albeit at considerably lower levels than in HeLa cells (see also, S1 and S2 Movies).

### Population deep sequencing of longitudinal samples identifies multi-step adaption to novel host environments

**Fig 3:**
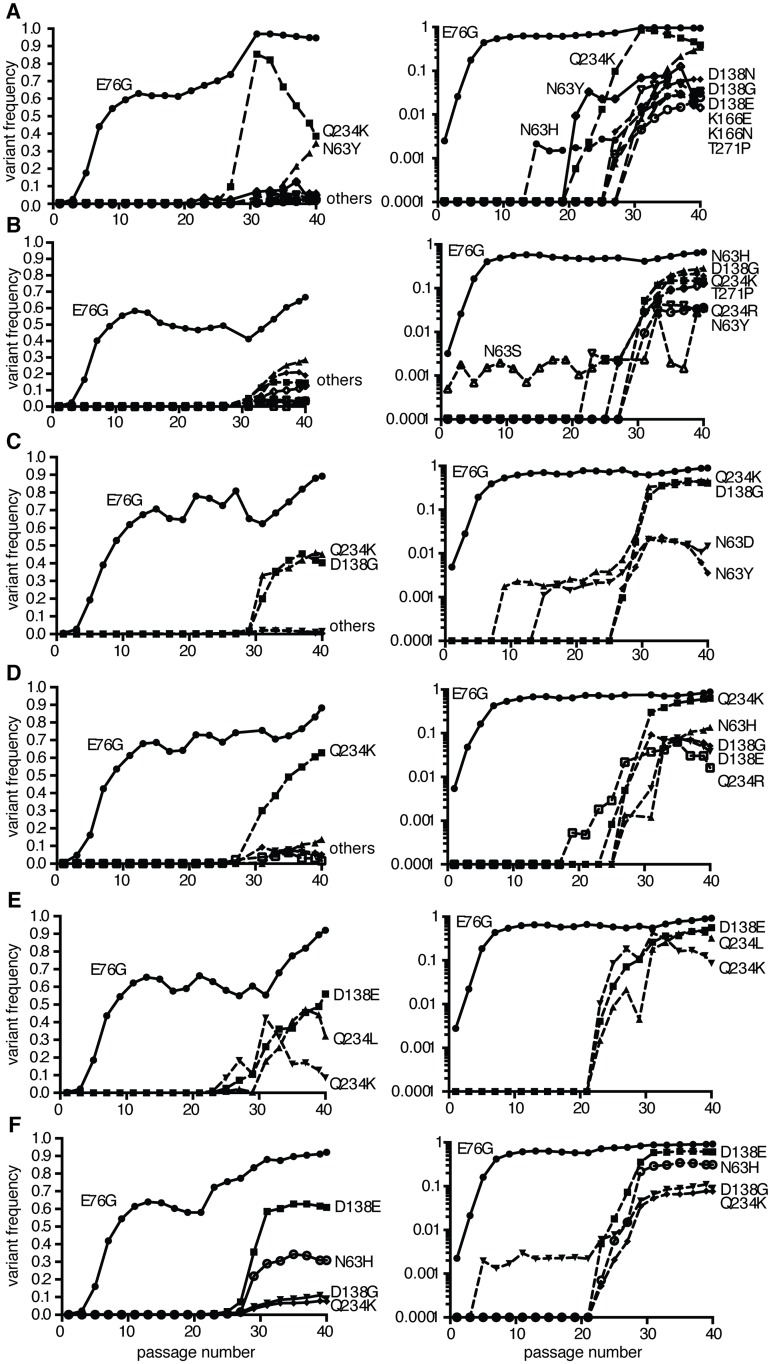
Population sequencing reveals multi-step adaptation of residues in the DAF-specific footprint during passage in A549 cells. (A-F) The virus populations were purified and deep sequenced for each replicate every two passages (p1, 3, 5, 7, etc...) across the P1 region coding for the four capsid proteins. The variant frequencies were determined by the ViVAN pipeline and all statistically significant mutations that showed a trend of increasing frequency were plotted in linear scale (left panels) and log scale (right panels). Each set of linear and log graphs represents the same data from one replicate, all six replicate passage series presented in this work are shown.

**Fig 4:**
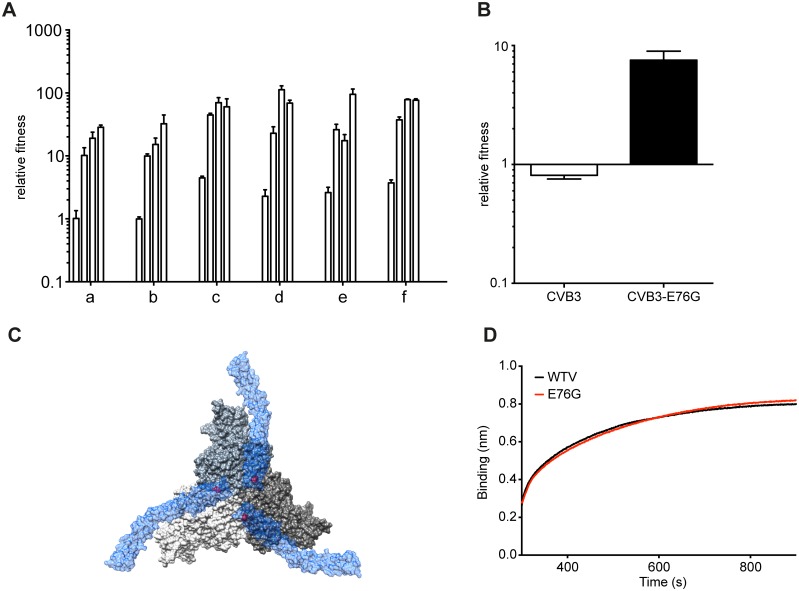
Mutation E76G confers fitness advantage in A549 cells. (A) Increasing relative fitness (y-axis) of replicates a-f, over the initial stage of passage series (histograms indicate values for passage 5, 7, 9, 11), mean values with SEM, n = 3. (B) Relative fitness of the CVB3-parental or E76G mutant generated from an infectious cDNA. The relative fitness (y-axis) is the ratio of the viral RNA quantification at 24h and 0h, mean values with SEM, n = 3. (C) The surface rendered map of three CVB3 protomers shown at the icosahedral three-fold axis of the virus (grey). Three symmetry related bound molecules of DAF (blue) are shown with a transparent surface rendering to allow visualization of the location of VP3 76 (red) on the virus surface directly below the DAF. (D) Binding to DAF was measured by biolayer interferometry by dipping a DAF loaded sensor into aliquots of CVB3-parental (black) or CVB3-E76G (red).

It has been shown that adaptation is often a multi-step process, especially in asexual populations like viruses. Usually, until the mutation with the largest beneficial effect becomes fixed, secondary beneficial mutations with less effect are unable to compete, thereby rendering the adaptation process sequential [17–19]. Given the different frequencies of minority variants observed at passage 40, we examined the patterns of emergence and sequential adaptation to novel environments by deep sequencing every second passage in the A549 cell series (Fig 3 and S1 Dataset). A number of step-wise trends were observed across the replicates. In all six replicates, the VP3-E76G mutation was the first mutation to emerge (already above 0.1% at passage 1) and peak by passage 11 to become the most abundant mutation in the population (between 65 and 95%). Furthermore, the increase in frequency of E76G over the first 11 passages (Fig 3) correlated with the increases in fitness over the same period (Fig 4A), underscoring a considerable contribution of this single mutation to population fitness. Indeed, the E76G variant was generated by reverse genetics and found to confer significantly enhanced fitness in A549 cells (Fig 4B). Although the VP3-76 residue was not previously identified as a contact between virus and receptor, this residue maps to the icosahedral three fold region of the capsid (Fig 4C). In the three dimensional virus-receptor structure, there is an interaction between the C-terminal 6-His tags of three symmetry related DAF molecules [20]. This interference by the His-tag may have restricted the normal interactions of DAF SCR3/4 with the virus surface, masking a potential role for residue 76. In a biolayer interferometry assay using BLItz technology [21] (Fig 4D), E76G bound DAF with the same affinity as wildtype virus, but no increased binding could be detected.

Despite the clear fitness advantage conferred by the E76G mutation alone (approximately 10-fold increase with respect to wildtype), it could not account for the more significant fitness increases observed in the passaged populations, particularly at late passage stages (nearly 100-fold increases). Following the fixation of this first mutation, the frequency of VP3-E76G plateaued until a second increase after passage 27, in each of the six replicates, coinciding with the emergence of different combinations of mutations, all mapping to the DAF footprint (Fig 3). Closer examination of the patterns of emerging mutations revealed potential synergistic and antagonistic epistasis among variants. Despite the advantage afforded by extreme depth in coverage, the Illumina technology used here set a limit of 69 nucleotides for read length. In principle, this limitation forfeits the possibility of linking more distant mutations and identifying haplotypes. However, the longitudinal deep sequence data revealed that several mutations increase with parallel kinetics and frequency, suggesting that: i) each mutation appeared in individual genomes and the variants were selected as a group and/or ii) the mutations are accumulating in the same genome, resulting in the selection of single haplotypes.

**Fig 5:**
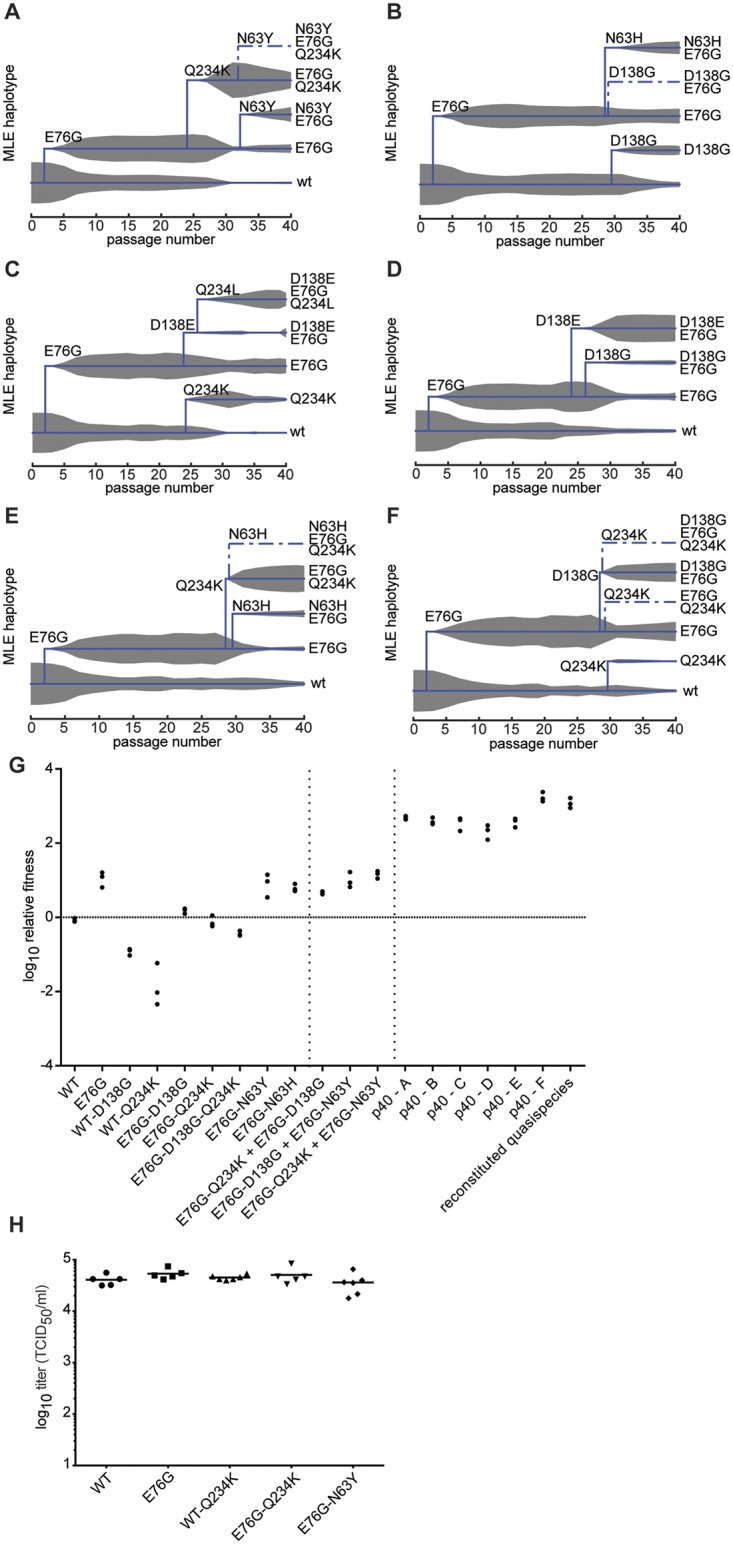
Maximum likelihood estimates of haplotypes present within the passaged virus populations and contribution of minority variants to population fitness. MLE was performed using the frequency values of each mutation at each passage in the deep sequence data for replicates a-f (A-F). The trees indicate the presence of wildtype genotype (lowest line on tree) along with the predicted haplotypes as they emerge during the passage series (x-axis). Solid lines indicate haplotypes with highest MLE scores, dashed lines indicate alternative haplotypes that could exist, with lower scores. The thickness of grey area indicates the expected frequency of each haplotype in the population, according to deep sequence data. (G) Relative fitness of the wildtype (WT), single, double and triple variants, followed by combinations of single variants, followed by passaged population samples (p40 replicates A-F) and reconstituted quasipecies. Reconstituted quasispecies is composed of 50:30:10:10 of E76G, E76G+N63Y, E76G+D138G, and E76G+Q234K. Vertical dashed lines are placed to separate the aforementioned groups to facilitate the reader. Horizontal line is placed to facilitate reading of neutral fitness (value 0). The relative fitness (y-axis) is the ratio of the viral RNA quantification at 24h and 0h, mean values with SEM, n = 3. (H) A549 cells were transfected with *in vitro* transcribed infectious RNA corresponding to dawildtype (WT), single and double variants. At 8 hours post-transfection, the progeny virus was quantified by TCID_50_ assay. No significant differences were observed between WT and variants (p = 0.203, 0.400, 0.365, 0.504, respectively, n = 5–6, two-tailed Mann Whitney test).

To distinguish between the above cases, phylogenetic trees describing the mix of haplotypes at each passage were inferred from the longitudinal data using maximum likelihood estimation in a Bayesian model. The best-fit, predicted haplotypes were then generated by reverse genetics and their relative fitness values were measured. As expected, this analysis illustrated the quick rise of the E76G genotype that continues to coexist with, although generally dominating over, the original WT genotype (Fig 5A–5F), with a significant increase in relative fitness (Fig 5G). For residue VP3-234, mutations were predicted to arise on either the WT background (Fig 5C and 5F) or the E76G genotype (Fig 5A and 5E). Mutations in residue VP2-138 were also predicted to arise on either WT (Fig 5B) or E76G (Fig 5C, 5D and 5F) genotypes at approximately the same time in the passage series as residue 234. However, their frequencies on the WT background tended to remain lower than on the E76G background where they seemed to be better tolerated. To confirm these modelled predictions, we generated each mutation on both backgrounds and measured the relative fitness. Indeed, the D138G mutation alone on the WT background conferred nearly a ten-fold drop in fitness and the Q234K mutation conferred up to 100-fold drop in fitness (Fig 5G), while the fitness costs of these mutations on the E76G background resulted in neutral fitness relative to WT. The data suggest that epistasis between these mutations and the E76G mutation rescues the fitness of these double variants and permits their positive selection in the viral population. Interestingly, the D138G and Q234K variants seemed to entirely exclude one another from the population (Fig 5A, 5B, 5D and 5E), and the MLE analysis suggested that if both are present in the population, they must occur on separate genomes. We thus generated the E76G-D138G-Q234K haplotype and confirmed that the triple mutant bears a significant fitness cost, rendering this haplotype less fit than the original WT genotype (Fig 5G).

Finally, shortly after the appearance of residue 138 and 234 variants, a third mutation appeared during the passage series at residue 63 (Fig 5A, 5B and 5E). Once again, computational modelling predicted that it too exists on the E76G background, and on separate haplotypes than the position 138 and 234 mutations. Furthermore, data from two replicates (Fig 5A and 5E) suggested that the E76G-N63Y double variant bears higher fitness than E76G-Q234K, as its frequency increased towards the end of the passage series as the other's decreased. Indeed, the residue 63 variants presented higher relative fitness than both the 138 and 234 variants on the E76G background (Fig 5G).

Although we could not determine whether residue 76 plays a role in receptor interaction directly, and the role of accompanying mutations are inferred from previously published studies, to rule out that these mutations impact virus fitness on activities downstream of receptor binding and entry, we transfected cells with *in vitro* transcribed RNAs corresponding to some of these variants and assayed virus production at 8 hours (before a new round of infection can occur). The data revealed that the fitness advantages (E76G, E76G-N63Y) and disadvantages (WT-Q234K, E76G-Q234K) observed during infection of cells (Fig 5G) do not appear to exist when the binding and entry step is bypassed (Fig 5H).

### Population fitness is determined by the group contribution of minority variants, rather than the selection of a dominant, single haplotype

Although we initially expected selection of fitness-increasing adaptive mutations to occur by step-wise accumulation of mutations on a single genotype, our computational analysis followed by fitness measurements of double and triple mutation-bearing genotypes suggested otherwise. Importantly, none of these single, double and triple mutations conferred the same fitness increases as those observed in the passage 40 virus population used to identify mutant composition (Fig 5G). Since previous work suggested that overall population fitness results from cooperative interactions among key variants in the mutant swarm, we examined whether a reconstituted composition of the most predominant variants within the passage 40 population could manifest comparable fitness. We thus generated artificial quasispecies presenting mixtures of position 63, 138 and 234 mutations on the E76G background and tested their relative fitness. Interestingly, artificial quasispecies presenting 63:138, 138:234 or 63:234 combinations at 1:1 ratios all presented fitness increases as high as, or higher than, the individual values for each variant (Fig 5G). On the other hand, none of these combinations resulted in the highest fitness values that were observed for the passage 40 populations of each replicate used to identify these individual mutations (Fig 5G, p40 a-f).

To address whether the minority variant composition may dictate observed population fitness, we reconstituted an artificial quasispecies based on the average frequency of each variant in passage 40 populations. In contrast to the individual variants or the 50:50 combinations described above, a mixture of 50:30:10:10 of the four most predominant genotypes E76G, E76G-N63Y, E76G-D138G, and E76G-Q234K, reproducibly reached the same fitness values as passage 40 samples themselves (Fig 5G), demonstrating that the global fitness of a virus population is determined by cooperative contribution of minority variants, rather than the dominant genotype alone.

## Discussion

**Fig 6:**
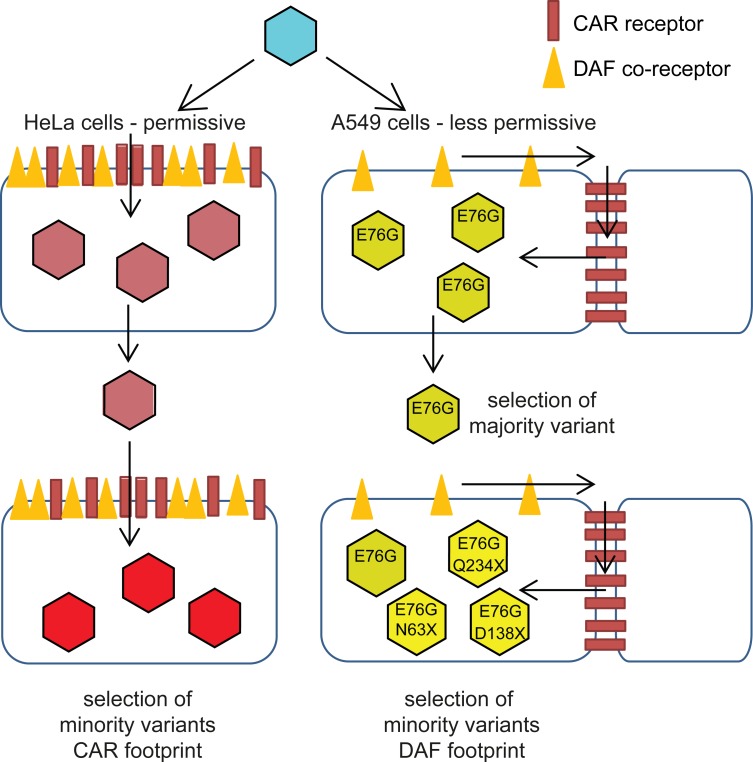
Schematic of CVB3 adaptation to differently permissive environments. In the permissive HeLa cell type, where the both CAR and DAF are highly and ubiquitously expressed, CVB3 accumulates CAR- and CAR/DAF-specific minority variants. In the less permissive A549 cells, where CAR is sequestered at cell-cell junctions and DAF is poorly expressed at the surface, CVB3 first fixates the E76G DAF-specific mutation, with the strongest single contribution to fitness, and in later stages, group selection of other DAF-footprint minority variants occurs.

Taken together, the sequence data and *in vitro* characterization of CAR/DAF expression provide a model for the adaptive dynamics of CVB3 to the two host environments presented here (Fig 6). In HeLa cells, where both CAR and DAF are highly and ubiquitously expressed on the surface, adaptive mutations mapped to both footprints. It is not clear whether the CAR-specific mutations (e.g. VP1-K259M) observed in HeLa cells increased interactions with CAR, or conversely, decreased interactions to facilitate the appearance of other mutations related to the DAF footprint. Because the particular strain of CVB3 Nancy used to initiate the passage series already contains some DAF-specific binding residues not found on other Nancy clones, we cannot speculate with confidence which way evolution would go in HeLa cells. In retrospect, inclusion of a CAR-exclusive binding strain of Coxsackie virus in the passage series would have helped discern the direction of evolution in HeLa cells. In A549 cells, on the other hand, the principal receptor CAR is mainly located in tight cell-cell contacts and likely inaccessible to the virus during initial stages of infection; while DAF is present throughout the surface, but at relatively low levels compared to HeLa cells. Thus, the focus of selection is entirely on residues involved in DAF binding (VP3-63, VP3-234) or shared in the CAR-DAF footprints (VP2-138). The CAR-specific mutations observed at residue VP1-259 in HeLa cells, were not observed in A549 cells. The dominant mutation, VP3-E76G, occurred in all six A549-passaged replicates and in none of the HeLa passages—a residue that was not identified as a determinant by conventional structural studies of receptor usage [20]. In the original structural studies, the interaction of residue VP3-76 could not be determined due to steric hindrance of the 6 His-tag between the DAF short consensus repeat domains, SCR3/4, and the virus surface. When CAR is not accessible, DAF is thought to facilitate translocation of the virus to tight junctions containing CAR [22,23]. The interaction with CAR mediates the transition to the A-particle, a required entry intermediate of expanded structure relative to native virus [24]. Nevertheless, it is possible that E76G improves the fitness of the virus in aspects not related to receptor binding, even if we could not identify these mechanisms when comparing replication cycles.

Our work reveals the power of deep sequencing to monitor the population dynamics of virus adaptation in new environments. By Sanger sequencing, only the E76G mutation would have been identified in each replicate. The remainder of mutants found to be positively selected during adaptation to host environment were all minority variants that could have otherwise been missed in multiple replicates. An issue inherent to deep sequencing technology is the estimated error of the chemistry (0.1% for Illumina sequencing), which has been regarded as a caveat to properly describe RNA virus quasispecies or mutant spectra. This problem was recently resolved by elegant molecular biology techniques to remove background error [8,25]. By applying more stringent bioinformatic treatment in our study, between 1700 and 2500 individual, statistically significant point mutations were identified in the structural protein region of the six replicates of A549-adapted populations; yet only mutations in positions related to the DAF footprint underwent selection and amplification over time (S1 Dataset). We thus show that robust bioinformatic treatment coupled with longitudinal data can circumvent error-related issues by identifying changes in variant frequency that would indicate positive or negative selection. This is particularly relevant to *in vivo* or clinical samples where the quantity of RNA genomes may be too low for more direct sequencing approaches and would require PCR amplification. Similar, relatively simple experimental studies could be designed to understand the population dynamics and evolution of pathogens in new environments during host adaptation and host switching, by identifying the selection over time of one or more mutations as single or multiple genotypes. However, one must keep in mind that although *in vitro* studies, such as ours, using immortalized cell lines may facilitate studying the dynamics of adaptation; but the specific mutations that are identified may not be indicative of the panel of mutations that would arise *in vivo*.

In this work, adaptation to a novel host environment was a multistep process involving the emergence of an initial mutation (E76G), followed by selection of a number of minority variants on the E76G background. Initially, we expected mutations to accumulate in a step-wise manner in a single genotype; however, fitness assays revealed that genotypes harboring double and triple combinations of these mutations presented fitness decreases relative to the wildtype and/or E76G backgrounds. Instead, computational inference of haplotypes suggested that such variants existed as a heterogeneous population of distinct genotypes. An intriguing observation was that no single variant (nor combination of two variants) conferred the high fitness values observed in the passage 40 populations. Strikingly, only the combination of the four most predominant genotypes, as an artificial quasispecies with the same frequencies observed in the passage 40 populations, was able to confer the same fitness values as the p40 replicate samples. This phenomenon is reminiscent of previous work that suggested that minority variants within an RNA virus quasispecies may contribute significantly to phenotype [26]. In that study, a high-fidelity poliovirus with restricted quasispeces composition was unable to infect the central nervous system (CNS); while the same virus stock that was chemically mutagenized to present wildtype numbers of minority variants restored the ability to disseminate to the CNS. Unfortunately, deep sequencing technology was not yet available and the authors could not uncover the identity of these presumed minority variants by Sanger technology to confirm their hypothesis. Here, we succeed in identifying the variants involved. Importantly, we provide evidence for group selection within the virus population and show that only the group contribution of these positively selected minority variants confers the fitness phenotype observed in the original samples. Our results thus illustrate the significant role of minority genomes in the fitness and phenotype of a virus population, providing further evidence for the quasispecies behavior of RNA viruses under certain conditions. It is important to note, however, that the group selection observed in this study resulted from relatively large population size in passages that reached high MOI by the time cell monolayers were lysed for subsequent passage. It is possible that in conditions where MOI is low, or when population bottlenecks occur (particularly *in vivo*), that the emergence of such minority variants would be delayed or impeded.

## Materials and Methods

### Cells, virus stocks and passages

HeLa and A549 cells (American Type Culture Collection) were maintained in DMEM medium with 10% new-born calf serum. Coxsackie virus B3 (Nancy strain) was recovered from a pCB3-Nancy infectious cDNA plasmid [27] that was linearized with Sal I and *in vitro* transcribed using T7 RNA polymerase. It should be noted that unlike other Nancy strains, this infectious clone already has VP2-138D and VP3-234Q residues, known to facilitate binding to DAF. 4 μg of transcript were electroporated into 4 x 10^6^ Vero cells that were washed twice in PBS and resuspended in PBS at 10^7^ cells/ml. For A549 cells, 15 μg of transcript were electroporated into 10 x 10^6^ cells. Electroporation conditions were as follows: 0.4mm cuvette, 950 μF, 250V, maximum resistance, exponential decay in a Biorad GenePulser XCell electroporator. Cells were recovered in DMEM-10% NCS. For each passage (40 passages total), virus was titrated by TCID_50_ and 400 μl of medium containing 2 x 10^5^ TCID_50_ was used to infect 2 x 10^6^ Hela or A549 cells in 6 well plates using a multiplicity of infection (MOI) of 0.1. Cells were incubated with virus for 45 minutes with frequent rocking, the supernatant was removed and monolayers were washed twice with 2ml PBS, then replenished with 2ml of complete medium. For each passage, virus was harvested at total cytopathic effect (CPE) by one freeze-thaw cycle, representing 2–3 viral generations. Six biologically independent stocks and passage series were generated.

### Viral titers by tissue culture infectious doses (TCID_50_)

Ten-fold serial dilutions of virus were prepared in 96-well flat-bottom plates in DMEM. Dilutions were performed in octuplate and 100 μl of dilution were transferred to 10^4^ Vero cells plated in 100 μl of DMEM-10% NCS. After 5 days living cell monolayers were colored by crystal violet. TCID_50_ values were determined by the Reed and Muensch method. No significant differences were observed between TCID_50_ values when using Vero, HeLa or A549 cells as the cellular substrate.

### Sequencing

5x10^8^ virion from passaged samples were RNA extracted and RT-PCR amplified by RT (Superscript III) and PCR (Phusion) using primers sets that covered the whole genome, in 3–4 kb fragments. For consensus sequencing, the resulting PCR products were purified, sequenced and analyzed using Lasergene software (DNAStar Inc). For deep sequencing, PCR fragments were purified via the Nucleospin Gel and PCR Clean-up kit (Macherey-Nagel) and total DNA was quantified by Nano-drop. PCR products were then fragmented (Fragmentase), linked to Illumina multiplex adapters, clusterized and sequenced with Illumina cBot and GAIIX technology. Sequences were demultiplexed by CASAVA with no mismatches permitted. Clipping was performed using the fastq-mcf tool, removing common adapter contaminants and trimming low quality bases (Phred<30). Clipped reads were aligned to the Coxsackie virus B3 Nancy sequence as reference with a maximum 2 mismatches per read, and no gaps, using BWA v0.5.9. Alignments were processed using SAMTools to obtain a pileup of the called bases at each position. An in-house pipeline, termed ViVAN (Viral Variant ANalysis) [28] was used to identify statistically significant variants above the background noise due to sequencing error, in every sufficiently covered site (>100x). Briefly, for each position throughout the viral genome, base identity and their quality scores were gathered. Each variant was determined to be true using a generalized likelihood-ratio test (used to determine the total number of minority variants) and its allele rate was modified according to its covering read qualities based on a maximum likelihood estimation. Additionally, a confidence interval was calculated for each allele rate. In order to correct for multiple testing, Benjamini-Hochberg false-discovery rate of 5% was set. The total allele rates passing these criteria, across the whole genome, were used to calculate the mean variation rates (diversity) at different passages. The variation rate at position i is defined as the proportion (F) of significant non-reference alleles (k) and is denoted V_i_:

$$V_{i}=\sum_{j=1}^{k}F_{ij}$$

The region-wide variation rate is the averaged variation rate across all covered positions in the genome (denoted n):

$$V=\sum_{i=1}^{n}\frac{V_{i}}{n}$$

### Confocal microscopy

Hela and A549 cells were plated onto coverslips, fixed with 2% paraformaldehyde for 20 minutes at room temperature, and then washed with PBS. Before staining, non-specific staining was blocked by incubating the cells with 5% FCS and 0.05% saponin in PBS during 10 minutes. Staining antibodies were diluted also in this buffer, and it was used for washes between antibodies. Cells were incubated with either CAR (Santacruz) or DAF (Abcam) primary antibody, washed, stained with a secondary antibody coupled to the appropriate fluorophore and washed. Cells were analyzed using a Zeiss LSM-700 confocal microscope. 3D reconstruction of the images was performed using the Imaris software.

### Western blot

One million cells of HeLa and A549 cells were lysed using a buffer containing 1% Triton-X and 1% sodium deoxycholate with protease inhibitors (Sigma). A fraction of the lysate was run for 1 hour on a 4–15% gradient gel (Biorad) on denaturalizing conditions. After the run, we performed the transfer to a nitrocellulose membrane. We washed the membrane with PBS-T (PBS 1X and 0.1% Tween-20) and blocked for 1 hour in PBS-T plus 5% milk. After the blocking we washed again with PBS-T and left overnight with each one of the antibodies, anti-CAR and anti-DAF (Santacruz Biotechnology). We washed the membrane and we added the secondary fluorescent antibodies (DyLight 680 and DyLight800 conjugated, Thermo Scientific) for 1 hour. We washed the membrane one last time and measure the fluorescence in the Odyssey system (Li-Cor).

### Flow cytometry

In order to prepare cells for cell cytometry analysis, cells were washed twice with PBS 1x and trysinized, washed again twice in PBS. Cells were stained for 30 minutes with either CAR-PE or DAF-FITC antibodies (Millipore and Abcam) on ice. Unbound antibody was discarded and cells were washed again with PBS 1x. Cells were resuspended in 1% Parafolmaldehyde (PFA, Electron Microscopy Sciences) and kept in the dark for 15 minutes. After the incubation PFA was discarded and cells were resuspended in 200 μl of PBS 1x. Cells were kept at 4C until analysed. For each cell type used (HeLa and A549) specific instrument settings were set according to the size and complexity of the cell type, as well as antibodies fluorescence. Samples were analysed using the MACSquant flow cytometer (Miltenyi Biotec) using 96 well plates and obtaining 10,000 events per sample. Mock samples were also used in each plate to setup the baseline. Results were analysed using Flowjo software v10.

### Fitness assays

Relative fitness values were obtained by competing each virus population with a marked reference virus that contains four adjacent silent mutations in the polymerase region introduced by direct mutagenesis. Co-infections were performed in triplicate at an MOI of 0.01 using a 1:1 mixture of each variant with the reference virus for 24 hours. The proportion of each virus was determined by real time RT-PCR on extracted RNA from the infection supernanant, using a mixture of Taqman probes labelled with two different fluorescent reporter dyes. MGB_CVB3_WT detects WT virus (including the fidelity variants) with the sequence CGCATCGTACCCATGG and labelled at the 5’ end with a 6FAM dye (6-carboxyfluorescein) and MGB_CVB3_Ref containing the four silent mutations: CGCTAGCTACCCATGG was labelled with a 5’ VIC dye. Each 25 μl-reaction contained 5ul of RNA, 900nM of each primer (forward primer, 5’-GATCGCATATGGTGATGATGTGA-3’; reverse primer, 5’-AGCTTCAGCGAGTAAAGATGCA-3’) and 150 nM of each probe. The relative fitness was determined by the method described by Carrasco et al. [15]. Briefly, the formula W = [R(t)/(R (0))] ^(1/t), represents the fitness, W, of each mutant genotype relative to the common competitor reference sequence, where R(0) and R(t) represent the ratio of mutant to reference virus densities in the inoculation mixture and t days post-inoculation, respectively. The fitness of the normal wildtype to reference virus was 1.019, indicating no significant differences in fitness due to the silent mutations engineered in the reference virus.

### BLItz assay

CVB3 parental strain, and CVB3-E76G were propagated in HeLa cells and purified as described previously [14]. The membranes of infected cells were broken by three freeze-thaw cycles. Virus was concentrated by pelleting through sucrose and purified by tartrate step gradient ultracentrifugation. The virus bands were collected and exchanged into PBS and virus concentration and quality was estimated by measuring the absorbance at 260, 280, and 310 nm. Binding assays were performed by biolayer interferometry (BLI) measured by the BLItz from Fortebio [21]. Briefly, BLI measures binding by sending white light down a glass fiber-based biosensor, which is reflected back up to the instrument from two interfaces: 1) the interface between the glass fiber and the biosensor, and 2) the interface between the surface chemistry and solution. Since the two reflections come from the same white light source in the instrument, they both contain the same wavelengths. When molecules bind to the surface of the biosensor, the path length of the reflection (the one reflecting from the interface between surface and solution) increases while that of the other reflection remains the same.0.4% BSA and 0.08% Tween was added to virus and DAF to prevent non-specific binding to the sensor during the assay. Purified, His-tagged DAF was diluted to 0.1 mg/ml and attached to a Ni-NTA sensor. The DAF loaded sensor was then dipped into virus diluted to 0.5 mg/ml.

### Mathematical modelling of emerging minority variants in deep sequence data

Let *X* be a phylogenetic tree, taken to include relative population sizes of the branches, and let *X*_*t*_ = (*s*_*t*_ *p*_*t*_)^*T*^ be the state of the tree at time *t* = 1, 2,…,*T*. Here *s*_*t*_ denotes the structure of the tree, i.e. the set of branches existing at time *t* and *p*_*t*_ is a vector with the relative population sizes of all existing branches. Furthermore, let *Y* be the set of measurements, where *Y*_*t*_ ∊ ℤ_+_^64*xN*^ is the number of reads supporting each of the 64 codons at each of the *N* different positions in the genome. By Bayes' theorem,

P(X|Y)∝P(Y|X)P(X)

The output probability P(*Y* | *X*) follows a multinomial distribution for each position. The dynamics of the phylogenetic tree are naturally assumed to be Markovian, i.e.

$$\text{P}(X)=\text{P}(X_{1})\prod_{t=2}^{T}\text{P}(X_{t}|X_{t-1}),$$

where the transition probability can be expressed using the structural and population dynamics parts separately,

$$\begin{array}{c} \text{P}(X_{t}|X_{t-1})=\text{P}(p_{t}|s_{t},s_{t-1},p_{t-1})\text{P}(s_{t}|s_{t}{}_{-1},p_{t-1})\\ =\text{P}(p_{t}|s_{t},p_{t-1})\text{P}(s_{t}|s_{t-1},p_{t-1}). \end{array}$$

A simple random walk model is used for the population dynamics P(*p*_*t*_ | *s*_*t*_, *p*_*t*-*1*_), the population change from *t*−1 to *t* is taken to be lognormally distributed with standard deviation *σh* where *h* is the length of the time step. The structural part, P(*s*_*t*_ | *s*_*t*−1_, *p*_*t*−1_), depends on the mutation frequency of the virus and the population size of the haplotype in which the mutation occurs. The posterior probability is thus, assuming that *s*_1_ and *p*_1_ are known,

$$\text{P}(X|Y)\propto \text{P}(Y|X)\prod_{t=2}^{T}\text{P}(p_{t}|s_{t},p_{t-1})\text{P}(s_{t}|s_{t-1},p_{t-1})$$

### Inference of haplotypes by maximum likelihood estimation

To make a maximum likelihood estimation (MLE) of *X* from the posterior tractable, some approximations are made. The attention is limited to a small set of variants, greatly reducing the number of possible tree structures. The most prevalent variants in the data should be included as they dominate the dynamic behaviour, but minority variants of particular interest can also be added.

Every possible tree structure matching the selected set of variants is generated. The time of appearance for each variant is set to where it is first seen in the measurements, i.e. the first time the frequency is above a small threshold. An MLE of P(*p*_*t*_ | *s*_*t*_, *Y*_*t*_) is then computed for each tree and time point. The rationale is that there is very little freedom for the population sizes to deviate from a point which can explain the output data. By construction of the tree structure, the number of non-reference haplotypes equals the number of variants at each time point in every tree. Hence, there is at most one convex combination of the haplotypes that match the event probabilities of the multinomial distribution for the output data. Due to the high number of reads in the deep sequencing data, moving away from the optimal point will cause a quick drop in the posterior probability. Dependencies between variants that are close enough to be covered by a single read are included in the model (amino acid residues 63 and 76).

The MLE of *X* can be found by evaluating the posterior for each generated tree and picking the most likely. All generated trees contain the same number of mutations. Since the value of the posterior probably only needs to be known up to a multiplicative constant, the effect of the overall mutation frequency of the virus on P(*s*_*t*_ | *s*_*t*−1_, *p*_*t*−1_) cancels out. Hence, P(*s*_*t*_ | *s*_*t*−1_, *p*_*t*−1_) is simply proportional to the population size of the haplotypes in which the mutations occur.

## Supporting Information

S1 Fig Expression of DAF in HeLa and A549 by confocal microscopy. Localization of DAF, in green, in HeLa (A) or A549 (B) cells by confocal microscopy. Nuclear staining was done with DAPI (blue). (B) DAF could only be detected when the exposure was increased 5 times compared to the HeLa settings.
(PDF)

S1 Movie Expression of CAR in HeLa cells. Hela cells were plated onto coverslips and processed for confocal microscopy analysis, as described in Materials and Methods. Z stack images acquired for Fig 2F and 2G were mounted as a video using the Imaris software.
(AVI)

S2 Movie Expression of CAR in A549 cells. A549 cells were plated onto coverslips and processed for confocal microscopy analysis, as described in Materials and Methods. Z stack images acquired for Fig 2F and 2G were mounted as a video using the Imaris software.
(AVI)

S1 Dataset Minority variants in replicate passage series in A549 cells. The following tables reveal all of the minority variants identified by bioinformatic analysis that are present at frequencies above 0.001 (0.1% of total population). The limit of detection where variants could not be distinguished from background error was always above 0.0001 frequency, but for clarity, variants at 0.0001 to 0.001 frequencies are not shown. The total number of variants in each population are quite similar, ranging from 1779 to 2005. The majority of these are very low frequency variants that do not change over the passage series. As would be expected by the generally neutral nature of synonymous mutations, nearly half of low frequency variants are synonymous. In some cases however, a number of synonymous mutations are found at increasing frequency from passage 1 to 40 (eg. nt. position 920 in replicate 4), or at significantly higher, yet relatively constant, frequency over the passage series (eg. nt. position 1283 in replicate 4). Their presence may indicate variants altering RNA structure, codon usage or other factors influencing fitness, but may also result from stochastic fixation of neutral mutations since they generally appear in only one of six replicates. Some variants with amino acid changes are present at considerable frequencies, without significant increase or decrease over the passage series (eg. VP1 position 104–106). Their contribution to the fitness of the population has not been studied here, but lack of increase in frequency suggests that they do not play a role in adaptation to A549 cells. Data from these tables was used to generate panels in Fig 4.
(XLSX)
