# Supplementary figures and images for: Group Selection and Contribution of Minority Variants during Virus Adaptation Determines Virus Fitness and Phenotype
Source: PLoS Pathog. 2015 May 5;11(5):e1004838. doi: 10.1371/journal.ppat.1004838 (PMC4420505; doi:10.1371/journal.ppat.1004838)

A

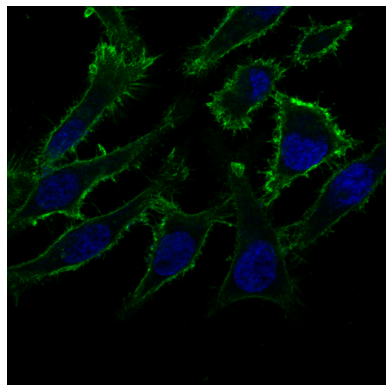

B

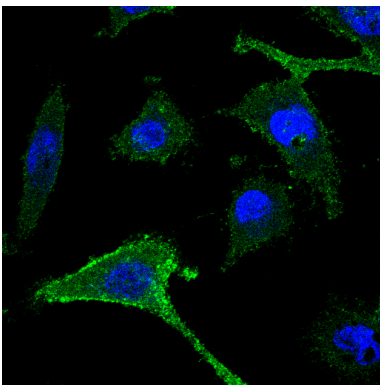

Supplement: S1 Fig — Localization of DAF, in green, in HeLa (A) or A549 (B) cells by confocal microscopy. Nuclear staining was done with DAPI (blue). (B) DAF could only be detected when the exposure was increased 5 times compared to the HeLa settings. (PDF) [file ppat.1004838.s001.pdf]
